# Implementation of piglet castration under inhalation anaesthesia on farrowing farms

**DOI:** 10.1186/s40813-022-00263-0

**Published:** 2022-05-17

**Authors:** Eva-Maria Winner, Marina Beisl, Sophie Gumbert, Helena Härtel, Jennifer Kaiser, Anja Wernecke, Steffanie Senf, Yury Zablotski, Mathias Ritzmann, Susanne Zöls

**Affiliations:** Clinic for Swine at the Centre for Clinical Veterinary Medicine, LMU Munich, Sonnenstrasse 16, 85764 Oberschleißheim, Germany

**Keywords:** Isoflurane, Anaesthesia, Piglet castration, Narcotic devices, Field study

## Abstract

**Background:**

Since 01.01.2021, suckling piglets may no longer be castrated without anaesthesia in Germany. Previous studies showed castration using isoflurane anaesthesia in combination with a suitable analgesic, meet the requirements of the German Animal Welfare Act. It can be carried out independently by farmers and other qualified persons with an automated and certified isoflurane device. Therefore, the aim of the present field study was to implement the use of three different anaesthetic devices for surgical castration of male piglets under automated isoflurane anaesthesia on 15 conventional pig farms in southern Germany. In addition, the depth of anaesthesia based on defensive movements, the labour time required in contrast to anaesthetic-free castration, castration-related anaesthetic incidents and the piglet mortality rate as well as occupational safety were investigated. For this purpose, farrowing batches of 11,574 piglets castrated under isoflurane anaesthesia (IA) were compared with the results of the 1568 piglets of anaesthetic-free farrowing batches (AF).

**Results:**

In total, 80.1% of the castrated piglets showed sufficient depth of anaesthesia, although this varied significantly between devices. 1.7% of the piglets suffered an anaesthetic incident, of which 0.1% died during or within 24 h after anaesthesia. The required time for the complete working process differed significantly between AF (1.7 ± 0.8 min/piglet) and IA batches (2.2 ± 0.8 min/piglet) but not for castration itself. The mean isoflurane consumption was 0.57 ± 0.27 ml/piglet and differed significantly between the devices (*p* < 0.001). The isoflurane concentration in the ambient air of the person-related workplace safety measurements was below the internationally lowest value of 15 mg/m^3^ from Ontario and Israel.

**Conclusion:**

In conclusion, 2 of the 3 types of devices used, a sufficient depth of anaesthesia during castration under isoflurane was achieved in 85% of castrated piglets. Anaesthetic incidents occurred in 1.7% of the animals, of which 0.1% died. Castration under isoflurane is more time-consuming than anaesthetic-free castration, but the castration time itself did not differ significantly. The occupational exposure limits were below the internationally lowest limit value of 15 mg/m^3^ for the persons involved. Even though castration under isoflurane is more time consuming than anaesthetic-free castration, it is a well-establishable method for practice and a dear improvement for animal welfare.

**Supplementary Information:**

The online version contains supplementary material available at 10.1186/s40813-022-00263-0.

## Background

There have been controversial discussions on the subject of the anaesthetic-free castration of male suckling piglets in Europe for years. Due to society’s growing interest in animal welfare, politicians had to make important decisions regarding this issue in recent years in Germany. The law prohibiting the castration of male pigs without anaesthesia came into force on January 01. 2021 [1]. Thus, piglet producers in Germany have the options to fatten boars with or without immunocastration or to castrate the piglets under anaesthesia and analgesia. Currently, two anaesthesia procedures of piglet castration fulfill the legal requirements of German Animal Welfare Act; on the one hand injection anaesthesia using ketamine and azaperone carried out by a veterinarian and on the other hand isoflurane inhalation anaesthesia can be performed by the farmers or other qualified persons themselves under certain requirements. They have to attend a 2-day general expert course and finally pass a written and an oral examination. Afterwards, a practical phase under the supervision of the veterinarian in charge of the farm and a successful practical examination on the anaesthesia device under the supervision of external examiners have to be carried out, in order to obtain the certificate [2]. There are currently around 7000 breeding sow farms in Germany [3]. In total, 2685 farmers received a state subsidy for their anaesthetic devices. This indicates, that roughly 40% of the sow farmers in Germany are currently castrating their piglets under isoflurane anaesthesia [4]. Presently, five certified anaesthesia devices from different manufacturers are approved by the German Agricultural Society (DLG) in Germany. Isoflurane is a frequently used volatile halogenated inhalation anaesthetic with a very good muscle relaxant and good hypnotic effect, but only a weak analgesic effect [5]. Therefore, it is indispensable to treat the piglets with a preoperative NSAID for 30 min before the castration procedure, to reduce pain afterwards [6]. In Switzerland piglets have already been routinely castrated by using isoflurane anaesthesia since 2010 [7] and in Germany, the method was already established on some organic farms. Several studies on isoflurane anaesthesia have already been carried out, which also depending on the narcotic device used, they came to different results with regard to the depth of anaesthesia. A previous study of Härtel et al. [8] was conducted under experimental conditions using automated isoflurane anaesthesia for suckling piglet castration on one piglet production farm. There, 955 piglets were castrated under isoflurane anaesthesia, of which 94% respectively 95% showed no or only a short defensive movement. Furthermore, only 0.9% of piglets suffered an anaesthetic incident such as apnea or cardiovascular arrest, but no piglet losses were recorded [8]. In the study of Enz et al. [7], data of 100 farms were analysed, whereof 86% of the castrated piglets showed no or only a short movement. Anyway, there are a few field studies reporting about insufficient anaesthetic depth during the piglet castration under isoflurane [9, 10]. Therefore, the aim of the present field study was to implement the use of three different anaesthetic devices for surgical castration under automated isoflurane anaesthesia on 15 conventional pig farms in southern Germany. In addition, the depth of anaesthesia based on defensive movements, the labour time required in contrast to anaesthetic-free castration, castration-related anaesthetic incidents and the mortality rate as well as occupational safety were investigated.

## Results

In total, data from 11,574 piglets from 129 farrowing batches in which castration was performed under isoflurane anaesthesia (IA) with three different devices (50 PigNap (PN) batches, 50 PorcAnest (PA) batches, 29 Anestacia (AN) batches) and data from 1568 piglets from 15 farrowing batches (AF) were collected, within these the castration process was still carried out without anaesthesia in 2020.

Due to repeated device failures, one IA batch in farm 2, 6, 11 each, five IA batches in farm 14, four IA batches in farm 7 and all ten IA batches in farm 10 couldn´t be analysed. In three farms (7, 10, 14) with AN device, it was not possible to perform castration due to undefined device failure despite support from the company's technical service. Problems with the evaporator (isoflurane concentration above 5 Vol % in the masks) led to increased occurrence of respiratory arrests in farm 2, 6 and 11. In farm 11, due to the high isoflurane concentration in the masks six piglets died during the castration procedure. Evaluation of the recordings showed a very short induction of anaesthesia followed by respiratory failure in all these animals. As a result, these batches were stopped and repeated after revision of the evaporator. Additionally, in farm 13, 69 animals showed shock conditions 10 min after castration under isoflurane anaesthesia. Of these animals, eight piglets died within 24 h after the castration procedure. Thereupon, iron was supplemented one day earlier, after that only isolated incidents occurred in IA batches but also in female piglets and were also reported earlier the context of iron supplementation. These animals were also excluded from the study.

## Anaesthesia incidents, mortality rate and defensive movements

**Fig. 1:**
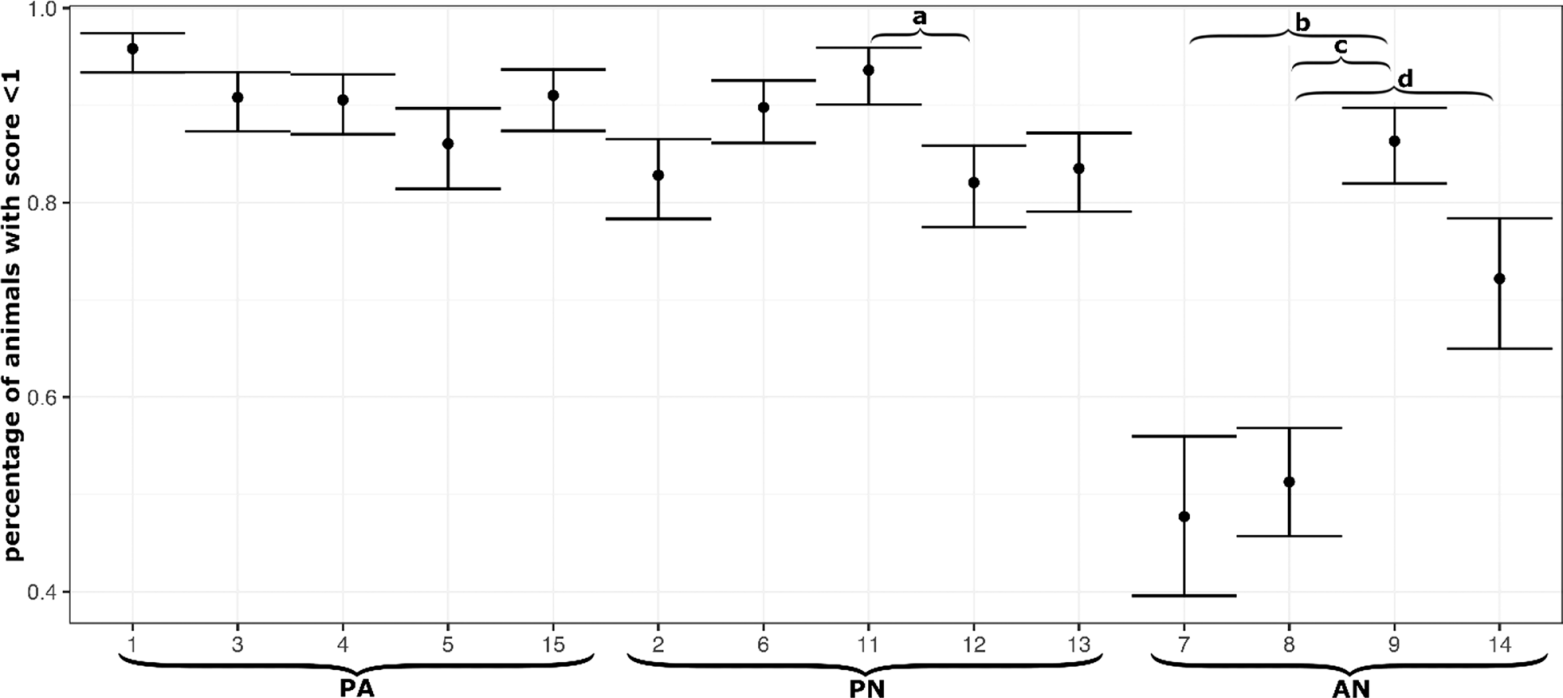
Percentage of animals with sufficient anaesthesia separated by farms (1–15, farm 10 excluded) (mean ± SD) and sorted by device (PA, PN, AN). Significant differences between farms within device groups are signed with small letters **a**: *p* < 0.0035, OR = 3.1629; **b** = *p* < 0.0001, OR = 0.1424, **c**: *p* < 0.0001, OR = 0.1655, **d**: *p* = 0.0007, OR = 0.4018

During castration process under isoflurane anaesthesia batches (IA), anaesthetic incidents such as apnea, cardiovascular arrest or gasp, were detected in 1.7% (n = 201) of all anaesthetised animals (Table 1). Within these 201 incidents, the most common anaesthetic incident was apnea with 66.7% regardless of the device type. Despite the introduction of appropriate countermeasures, the total mortality rate of the evaluated 129 batches, when castration process under anaesthesia were carried out without disruptions, was 0.1%. Three piglets (0.03%) died during castration under anaesthesia (all group PA) and eight piglet losses (0.07%) were detected within 24 h after castration (3 PN, 3 PA, 2 AN). In the AF batches (in the year 2020) six piglets died within 24 h after castration (mortality rate 0.4%). In total, 80.1% of the 11,574 evaluated animals showed sufficient anaesthesia during castration under IA with movement score 0 and 1 (Table 1). Moreover, 4.8% reacted with two short movements and 15.1% of the 11,574 piglets showed repeated movements (score 3 and 4) during isoflurane anaesthesia (IA). The percentage of piglets with sufficient anaesthesia differed significantly between devices (PN–PA: OR = 0.649, *p* = 0.0122; PN–AN: OR = 2.730, *p* < 0.0001; PA–AN: OR = 4.206, *p* < 0.0001) and farms. In AN Group, especially in farm 7 and 8, a very low percentage of sufficient depth of anaesthesia can be seen compared to the others (p < 0.05) (Fig. 1) The location of the device (stable aisle or farrowing compartment) had no significant effect on the depth of anaesthesia (*p* > 0.05).

**Table 1:** Overview of defensive movements and anaesthetic incidents divided after device

		Defensive movement score					Anaesthetic incidences		
Device	n	Sufficient n (%)			Insufficient n (%)		Total n (%)		
		0	1	2	3	4	apnea	cardio	gasp
PN	5128	4042	306	257	193	330	11	5	3
		**4348 (84.8)**		(5.0)	**523 (10.2)**		**19 (0.3)**		
PA	3526	3135	124	109	51	107	64	18	18
		**3259 (92.4)**		(3.1)	**158 (4.5)**		**100 (2.8)**		
AN	2920	1434	232	187	294	773	59	10	13
		**1666 (57.1)**		(6.4)	**1067 (36.5)**		**82 (2.8)**		
Total n (%)	11,574	8611	662	553	538	1210	134	33	34
		**9273 (80.1)**		(4.8)	**1748 (15.1)**		**201 (1.7)**		

The numbers in bold are the total result of the numbers added above

n = number castrated piglets

PN = PigNap, PA = PorcAnest, AN = Anestacia

Cardio = cardiovascular arrest

Score 0 + 1 = sufficient anaesthesia

Score 3 + 4 = insufficient anaesthesia

## Labour time

The required working time for the complete castration process per piglet varied significantly between anaesthetic-free (1.7 ± 0.8 min) and isoflurane anaesthesia batches (2.2 ± 0.8 min) (*p* = 0.012) (Table 2). The total preparation and post-processing time also differed significantly between AF and IA batches (*p* < 0.001), unlike the required time for the castration procedure per piglet (*p* > 0.05). Separated by device, the time per piglet for the castration itself, varied between 0.97 ± 0.38 (PN, n = 55 batches), 1.01 ± 0.28 (PA, n = 55 batches) and 1.07 ± 0.37 min (AN, n = 33 batches) without differing significantly (p > 0.05).

**Table 2:** Mean value of the required working time in minutes for single process steps and complete process

Required time (in minutes)	AF batch (n = 14)	IA batch (n = 45)		
	mean ± SD	min/max	mean ± SD	min/max
Preparation (total)	2.8 ± 1.6	1–6	17.3 ± 8.9	5–37
Analgesia (per piglet)	0.7 ± 0.4	0.1–1.6	0.5 ± 0.3	0.1–1.1
Castration (per piglet) *	0.9 ± 0.4	0.4–2.0	1.0 ± 0.3	0.3–2.0
Post-processing (total)	4.4 ± 3.7	1–14	21.1 ± 7.4	5–40
Complete process (per piglet)	1.7 ± 0.8	0.9–3.6	2.2 ± 0.8	0.8–4.6

IA = isoflurane anaesthesia

AF = anaesthetic free

^*^IA = 129 batches

The workload for the complete castration process varied between 3.0 ± 1.6 min per piglet (min: 1.5; max: 7.2) in AF and 4.5 ± 1.7 min per piglet (min: 1.5; max: 9.1) in IA batches (p = 0.002) (Additional file 1: Table S1).

## Isoflurane consumption

**Fig. 2:**
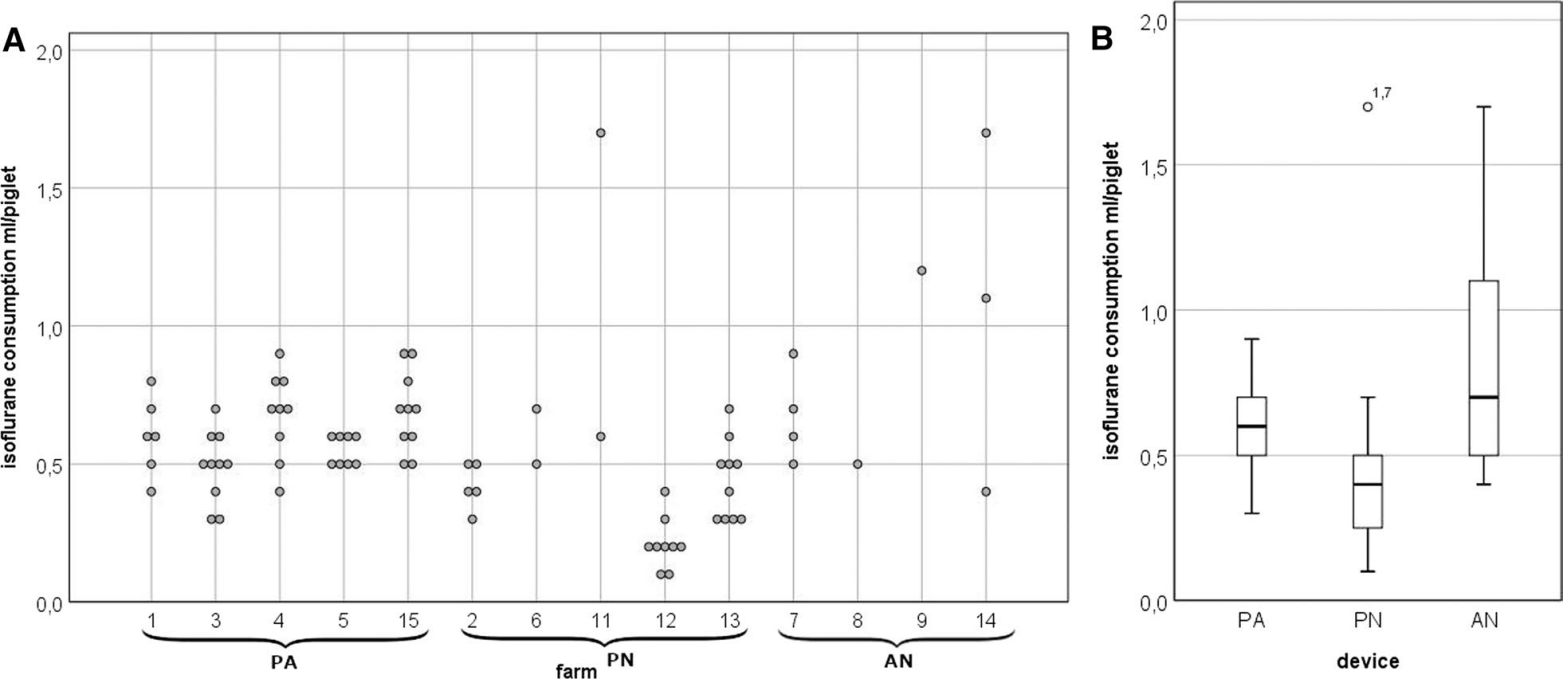
**A** Calculated isoflurane consumption (ml/piglet) per batch (o) separated by farm and device. **B**: boxplot of isoflurane consumption (ml/piglet) per device (PorcAnest (PA), PigNap (PN), Anestacia (AN))

Isoflurane consumption was calculated based on the used isoflurane in 80 batches (28 PN, 43 PA, 9 AN). The mean consumption in all evaluated batches was 0.57 ± 0.27 ml/per piglet and differed significantly between the devices (PN: 0.43 ± 0.30 ml/piglet, PA: 0.61 ± 0.13 ml/piglet and AN 0.83 ± 0.42 ml/piglet, *p* < 0.001) (Fig. 2). Again, the isoflurane consumption on farm 10 couldn´t be evaluated due to technical problems with the device.

## Isoflurane exposure

**Fig. 3:**
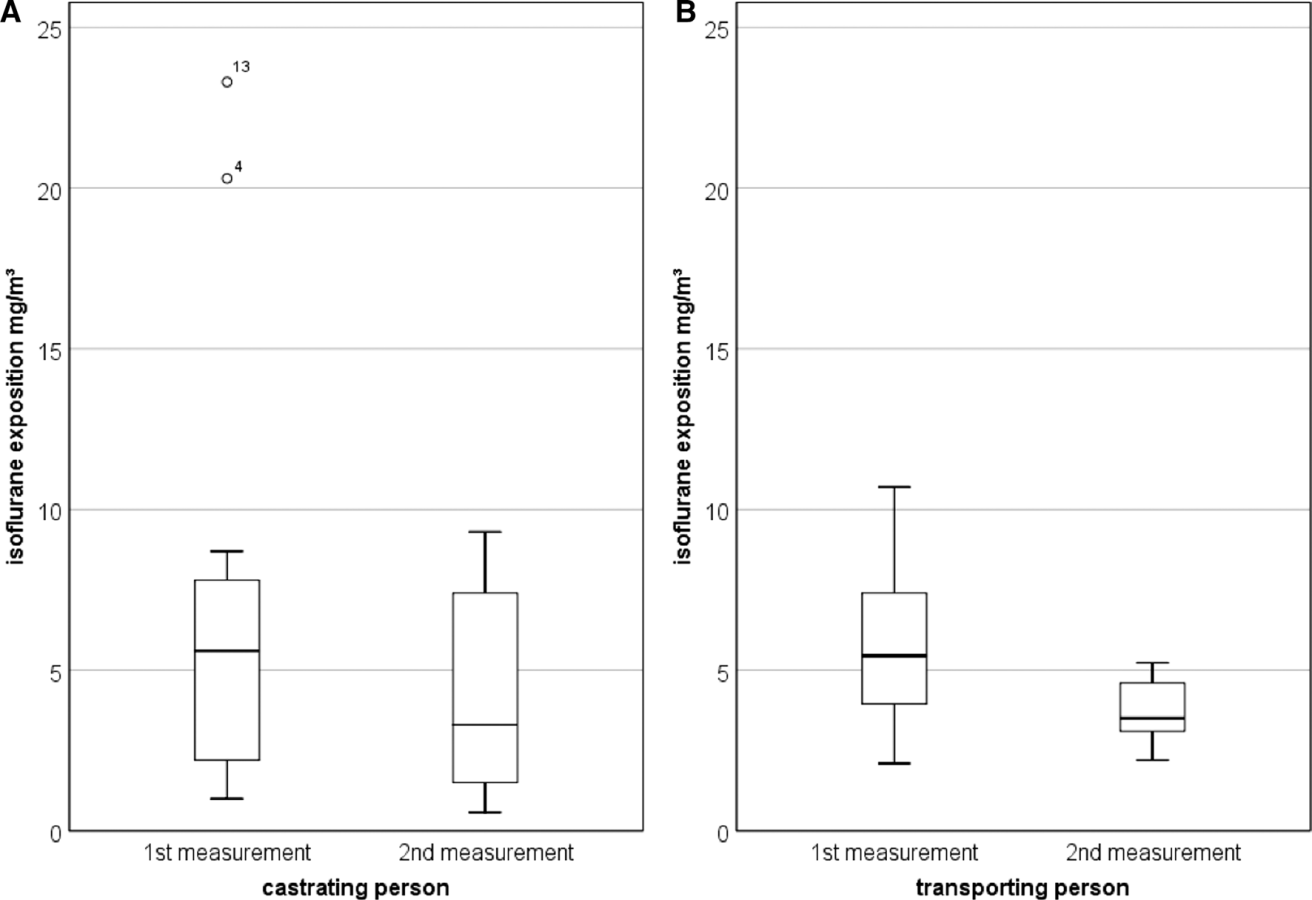
**A**: Boxplot of isoflurane exposition in the breathing air of persons castrating and **B**: transporting the piglets divided into first and second measurement

**Fig. 4:**
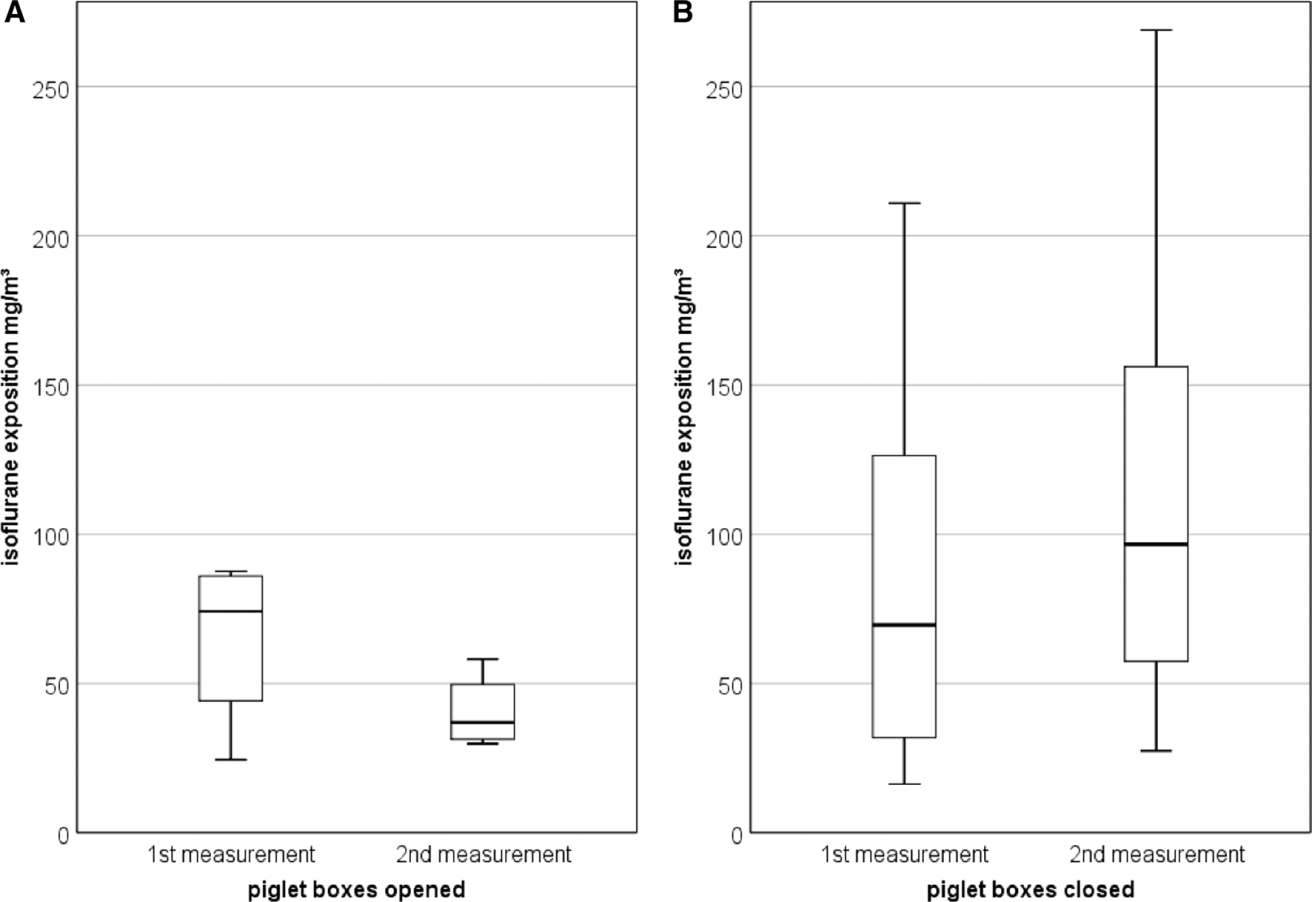
**A**: Boxplot of isoflurane exposition in opened piglet boxes and **B**: in closed piglet boxes

The isoflurane concentration in the ambient air was examined at five defined measuring points in 26 farrowing batches (Additional file 2: Table S2). No significant differences between the devices could be detected (*p* > 0.05). The mean concentration in the respiratory area of the castrating person was 5.8 ± 5.5 mg/m^3^ (1st + 2nd measurement). The person transporting the piglets was exposed to an average isoflurane concentration of 4.8 ± 2.2 mg/m^3^ (1st + 2nd measurement) The median of all measurements has decreased between the first and second measurement (Fig. 3). This is due to individual improvements (opening windows, doors) on the farms. In the area of the anaesthetic masks a value of 34.6 ± 29.7 mg/m^3^ was determined and the measurements at the respective activated carbon filters showed a mean value of 7.4 ± 11.5 mg/m^3^. Regardless of the piglet box type, a mean value of 88.2 ± 67.9 mg/m^3^ was calculated. The mean isoflurane concentration for first and second measurement, in opened and closed boxes is shown in Fig. 4 (52.8 ± 24.6 mg/m^3^ and 104.0 ± 75.4 mg/m^3^, respectively).

## Discussion

The aim of the present study was to evaluate the implementation of surgical castration under automated isoflurane anaesthesia with three different anaesthetic devices on 15 conventional swine farms in Germany. Animal welfare aspects, practicability, feasibility, cost-effectiveness, and occupational safety of isoflurane anaesthesia was investigated by using defensive movements, piglet losses, labour time, isoflurane consumption, isoflurane concentration in the ambient air and at critical points such as the inhaled air of the persons involved. Therefore, data of 11,574 piglets (129 farrowing batches), castrated under isoflurane anaesthesia were evaluated. The depth of anaesthesia was determined using a modified score of Wenger et al. [11] and Härtel et al. [8] based on defensive movements. Based on Härtel et al. [8], the scores 0 to 1, regarding defensive movements and vocalization, were defined as sufficient depth of anaesthesia. Whereas score 3 and 4 were defined as insufficient depth of anaesthesia. In our studies, sufficient depth of anaesthesia was observed in 80% of all castrated piglets, regardless of the anaesthetic device. Comparable results under similar conditions were observed in the swiss study of Enz et al. [7] where 86% of the piglets showed no or only one short movement. With the PA 92% (n = 3259) of the castrated piglets showed no or just one short movement (score 0 + 1). The study conducted by Härtel et al. [8] revealed similar results. Using the same device, 94% of the castrated piglets showed a sufficient depth of anaesthesia in this study. Also, earlier studies of Kupper et al. [12] reported in 92% of the castrated piglets no or just a short defensive movement. In the present study, one out of five PA devices experienced a technical problem during one batch. This was manifested by frequent respiratory arrest of the piglets in the device. Thereupon, the castration process was stopped until the problem was solved. With the PN, sufficient depth of anaesthesia was achieved in 85% (n = 4348) of the castrated piglets. These results differ from Schwennen et al. [13] and Steigmann et al. [9] who documented only 77% and 66%, respectively, of the monitored piglets showing a sufficient depth of anaesthesia. One reason for the higher results in our study is probably the technical revision of the devices, in respect to the German Agricultural Society (DLG) certification process, in 2020 [14]. Nevertheless, technical problems with the evaporator (high isoflurane concentrations on the masks) occurred with this device on three farms (2, 6, 11) which led to an increased rate of anaesthetic incidents. At farm 6, respiratory arrest was detected in four out of 35 piglets (11.4%) at the beginning of one batch. As a result, the castration process was stopped and the isoflurane concentration was measured on the masks using the vamos device from the company Dräger thereby a concentration of 8 Vol % was detected. Due to the quick introduction of appropriate measures by the farmer, none of the animals died. These incidents show that an increased concentration of isoflurane can lead to multiple occurrence of anaesthetic incidents. 57% (n = 1666) of the piglets castrated with the AN device, reacted with no or one light movement. A possible explanation for this low value, compared to the other devices, were persistent technical and management problems which could not be solved despite repeated involvement of the manufacturer. Therefore, not all farrowing batches on farm 7, 10 and 14 could be analysed. The main reason for the insufficient depth of anaesthesia on these three farms, was the low isoflurane concentration of less than 5 Vol % measured in the masks. The DLG report confirms the findings of the present study with regard to measured isoflurane concentrations in the masks of the AN device [15]. If the evaporator is set at 5 Vol % anaesthetic gas mixture, inspiratory concentrations of 3.7–4.3 Vol % are achieved depending on the ambient temperature and using all stations [14]. Management problems could be another reason for insufficient depth of anaesthesia. Video records showed that some piglets were already in the awakening phase although castration had not started yet. To prevent this, farmers should clamp fewer piglets in the operation units or work with a second person and if necessary extend the gas supply in time. Before castration is performed, the farmer must ascertain that the piglet is in a sufficient depth stage of anaesthesia [2]. As well as Härtel et al. [8] and Rintisch et al. [16], we used the interdigital claw reflex as a suitable tool to verify the surgical tolerance stage. To conclude, it is very important that the anaesthetic devices work reliably to ensure a sufficient depth of anaesthesia. In total 1.7% (n = 201) of 11,574 castrated IA piglets suffered from anaesthetic incidents, mostly in form of apnea followed by cardiovascular arrest and gasp. In contrast, Härtel et al. [8], reported a total of 0.9% incidents during or at the end of the castration procedure with the PA device. Explanations for this could be, that in the mentioned study a health score was collected from each piglet by veterinarians and only healthy piglets were castrated which reduced the risk for incidents. In addition, these piglets were always castrated after the standardised induction time of 90 s. Wenger et al. [11] recorded no anaesthetic incidents during their trial under halothane anaesthesia. In the present study the fewest anaesthesia incidents were observed with the PN device. Possibly due to the shortened introduction time of 70 s it was observed in 0.3% (n = 19) of 5128 castrated piglets. Regardless of the device, piglets suffering from apnea was the most common and frequent cause for interruptions during the castrating process in the entire study period. The total mortality rate of IA batches was 0.1% (n = 11). Within these, 3 piglets died during IA and 8 piglets died within 24 h after the procedure. Enz et al. [7] validated a similar mortality rate of under 0.1%. Prior studies reported no mortality within 24 h after castration [8–10, 17–20]. In the AF batches, six out of 1568 piglets (0.4%) died 24 h after castration, due to smothering by the sow. Comparison of the results (AF and IA batches) under the present conditions shows that if anaesthesia under isoflurane is performed correctly no increased losses should occur. Close observation of the piglets during the recovery phase must be ensured as common routine and if necessary, timely appropriate countermeasures have to be initiated. Particularities were observed at one farm, where 69 piglets suffered from a shock. These incidents occurred five to ten minutes after the castration process under isoflurane. At the time of seizure, piglets were awake and already able to stand and walk. Unfortunately, eight piglets suffering this shock died 24 h after castration. Thereupon, the internal procedures were changed so that iron administration was performed the day before castration. After modifying the routine, only isolated incidents occurred. Whether these shock states are related to the anaesthesia or whether the isoflurane only serves as a trigger for this process cannot be said at present. Genesis of the shock remained unclear, further investigations are still pending.

In this study, data regarding required working time and the workload was calculated. The required working time does not include the number of people involved, and exceeded for the complete process (preparation, analgesia, castration, post-processing) in IA batches (2.2 ± 0.8 min/piglet), the time in AF batches (1.7 ± 0.8 min/piglet) significantly. However, the time needed for the castration itself differed less strongly between IA and AF batches than complete castration process. This underlines, that the number of piglets per farm has to be considered when comparing the time for the complete castration process between farms or devices. These findings are similar to the study of Scollo et al. [21] they calculated an additional time in the analgesia/anaesthesia groups of 13:21 and 11:49 min compared to the control group without analgesia and anaesthesia of 7:33 min for each litter. In the study of Weber et al. [22] the mean required time for the complete process was 2.9 ± 0.3 min/piglet. Authors of earlier studies already determined an increased time required regarding the preparation under isoflurane anaesthesia. For instance Weber et al. [22] and Raaflaub et al. [23] calculated 28 and 20 min, respectively for setting up the PN device. Once farmers have developed a routine in using the anaesthetic equipment, the working time required per piglet for the castration did not differ significantly between AF (0.9 ± 0.4) and IA (1.0 ± 0.3) batches (*p* > 0.05), similar to Weber et al. [22] and Hodgson [18]. Walker et al. [20] estimated a mean of 2 min/piglet for the total anaesthesia and castration time required, regardless of an induction time of 90 resp. 60 s under experimental conditions. In the present study, the amount of time for castration and application of an analgesic per piglet was 1.5 ± 0.6 min in the IA batches, comparable to the mean of 1.2 min/piglet additional working time measured in former studies [22, 23]. Considering the number of people involved in the working process, the workload for the whole process increased to 4.5 ± 1.7 min/piglet in IA batches (Additional file 1: Table S1), and is in line to Enz et al. [7] and 3.0 ± 1.6 min/piglet in AF batches. Since isoflurane is a colourless halogenated ether, a maximum vapor concentration of 31.5% is achievable at 20 °C [5]. Like all anaesthetic gases, isoflurane is mainly exhaled and not metabolished [5]. For this reason, the survey of isoflurane consumption is not only interesting from an economic point of view, but also in relation to the environment and thus to occupational safety. In the present study the mean isoflurane consumption per batch was 0.57 ± 0,27 ml/piglet and varied between a minimum of 0.15 ml to a maximum of 1.7 ml/piglet (Fig. 2A). The highest consumption was detected in farms 11 (PN) and 14 (AN). The high value in farm 11 is due to technical problems with the evaporator which occurred after this batch. After fixing the problem, the consumption of the device was comparable with the other PN devices. The high consumption in farm 14 after the first batch probably occurred for the following reason: The evaporator was not yet completely filled with enough isoflurane at this point and thus more isoflurane was consumed. The significant difference regarding isoflurane consumption between the devices (PN 0.43 ± 0.30 ml/piglet, PA 0.61 ± 0.13 ml/piglet and AN 0.83 ± 0.42 ml/piglet) (*p* < 0.001) (Fig. 2B) could be caused by the frequent occurrence of technical problems mentioned earlier. Comparing the results with the data of the DLG, the measured consumptions of the PN are within the stated range, for the PA slightly above and for the AN considerably higher [14]. Regarding the results of the AN device, the few measurements, the single high values and the persistent problems with the devices have to be considered. Therefore, further measurements under field conditions with reliably functioning devices are necessary. The study of Enz et al. [7] from 2013 calculated a mean value of 0.87 ml ± 0.21 ml/piglet. One explanation for the lower consumption in the present study could be that, all devices have been checked and technically improved in recent years.

In cooperation with the TÜV SÜD Industry Service GmbH the isoflurane concentration in the ambient air was measured twice at each farm during two different batches but under similar conditions. Two farms had to be excluded due to persistent technical problems (farm 7, 10). Since there is no recommended work station limit for isoflurane in Germany yet, the working limit that should not be exceeded are the lowest limits that are applied worldwide. This corresponds to 15 mg/m^3^ from Ontario and Israel followed by the limit of 80 mg/m^3^ in Austria, Switzerland and Sweden [24]. In this study, the mean results of the person related-measurements were below the lowest work place limit of 15 mg/m^3^ from Ontario and Israel. In the ambient air of the person transporting the piglets, a mean concentration of 4.8 mg/m^3^ (Fig. 3B) was assessed whereas the castrating person was exposed to an average of 5.8 mg/m^3^ (Fig. 3A). These findings are in line with the results of Härtel et al. [8]. In the present study, at two farms (4, 15) elevated limits were detected in the ambient air of the castrating person probably due to lack of air circulation. Thereupon, more air velocity was provided for the next measurement by opening a door or window. In both cases, values of the second measurements were under the limit of 15 mg/m^3^ (Additional file 2: Table S2). In the area of the anaesthetic masks a value of 34.6 mg/m^3^ was determined. Similar to the investigations of Härtel et al. [8] and Säre et al. [25], we assume that the masks did not properly enclose the trunks of some animals. Consequently, this could lead to relevant concentrations in the ambient air and increased exposure for the persons, this isoflurane is not absorbed by the filters. The results at the respective activated carbon filter yielded a mean value of 7.4 mg/m^3^. At the different piglet boxes a mean value of 88.2 mg/m^3^ was calculated. In fact, in almost all farms the highest results were achieved in the piglet boxes. One explanation for this could be the different types of awakening boxes. Since in this study higher results were detected in closed boxes and lower values in opened boxes such as baskets with holes, the type of recovery boxes could have an influence on the distribution of isoflurane. In addition, it should be considered that the higher isoflurane concentration might lead to a prolonged post-sleep period due to the piglets' rebreathing. The values in the piglet boxes are primarily due to exhalation by the piglets, which is why retrofitting of the awakening boxes should be considered. Currently, there is one company in Germany, that offers a gas suction for the piglet box which might solve the problem. Although isoflurane contributes to the destruction of the ozone hole and promotes the greenhouse effect, inhalation anaesthetics are considered to be of little climatic importance [12]. In the study of Enz et al. [7], isoflurane concentration in the ambient air was 18.7 mg/m^3^. Kupper et al. [12] reported isoflurane concentrations at the castrating person and anaesthetic device of 3.7 mg/m^3^ and at the piglet boxes a concentration of 18.7 mg/m^3.^. Measurements of the social insurance for agriculture, forestry and horticulture (svlfg) exceeded in 11 out of 15 measurements with one device the 15 mg/m^3^. With a different device, 18 out of 18 measurements fell below the lowest limit of 15 mg/m^3^ [26]. Since these results were published before the devices were revised and certified in 2020, this could be an explanation for the different results. This indicates, that it is most important for work place safety, to perform castration under isoflurane anaesthesia in a well-ventilated environment (air exchange rate 3–5 m/sec.) [24, 27]. In addition, the devices should be regularly maintained and checked for function by the manufacturers in order to detect possible defects at an early stage [7]. As soon as farmers notice health complaints, the castration process must be stopped and the device checked by a professional technician. A clear limitation of the present study, is that not all three types of units could be tested at one farm in order to avoid the company effect.

## Conclusion

In summary, 2 of the 3 types of devices used, a sufficient depth of anaesthesia during castration under isoflurane was achieved in about 85% of the castrated piglets but varied considerably between device types and also farms and farrowing batches. Therefore, the results of the present study underline that each device must be frequently and individually checked with special regard to its animal- and user-safe function. The anaesthetic incidents of 1.7% and the mortality rate of 0.1% were comparable low to anaesthetic-free castration. Measurements of the isoflurane concentration in the ambient air of the castrating persons reached very satisfactory values constantly within the lowest available working place limit. For this, it is important that castration environment is well ventilated and that the devices are inspected regularly. Even though castration under isoflurane anaesthesia is more time-consuming, it can be a well-established method in practice, which if carried out correctly, is associated with low risks for the piglets and a dear improvement for animal welfare.

## Methods

The aim of the present study was to evaluate the implementation of surgical castration under automated isoflurane anaesthesia with different anaesthetic devices on 15 conventional farrowing farms of different sizes and farrowing rhythms (Table 3 Overview of the farms, castrated animals and evaluated farrowing batches (batch) separated by device) in Southern Germany. Male piglets on these farms were castrated according to German Animal Welfare Law under the age of 8 days with analgesic treatment by the farmer; before 2021 without anaesthesia and afterwards under isoflurane anaesthesia [2]. The evaluation of castration procedure was conducted under field conditions during routine animal management and procedures from September 2020 to July 2021. The castration process is defined as “batch”. Therefore, depth of anaesthesia based on defensive movements of the castrated piglets, castration-related anaesthetic incidents and the piglet mortality rate as well as occupational safety and the required working time, were investigated.

**Table 3:** Overview of the farms, castrated animals and evaluated farrowing batches (batch) separated by device

Device	Farm (ID)	Farm size	n piglets (n batches)		Isoflurane measure (batch ID)	Comments
			AF	IA		
PN	2	100–250, 3 wks, 102, ear tag	158 (1)	964 (10)	2,7	Repetition of batch 1, technical problems with the evaporator
	6	100–250, 3 wks,101	30 (1)	373 (10)	3,10	Repetition of batch 6, technical problems with the evaporator
	11	< 100, 3 wks, 38	42 (1)	382 (10)	4,9	Repetition of batch 4, technical problems with the evaporator
	12	100–250, 3 wks, 94, vaccination, ear tag	85 (1)	951 (10)	5,9	
	13	> 250, 3 wks, 243, ear tag, (iron)	223 (1)	2458 (10)	5,9	
PA	1	100–250, 3 wks, 101, iron	93 (1)	1019 (10)	4,8	
	3	> 250, 1-1-0, 74, tail dock	73 (1)	743 (10)	4,8	Repetition of batch 4, technical problems with the evaporator
	4	100–250, 3 wks, 82, iron	73 (1)	829 (10)	5,7	
	5	< 100, 3 wks, 47	66 (1)	456 (10)	5,8	
	15	< 100, 2 wks, 49, iron	64 (1)	479 (10)	7,8	
AN	7	> 250, 3 wks, 371	432 (1)	1424 (4)	1,3	No evaluation of batch 5,6,7,8,9,10 due to technical problems with the device
	8	100–250, 3 wks, 86, ear tag	127 (1)	819 (10)	5,8	
	9	100–250, 3 wks, 52, tail dock, iron	30 (1)	552 (10)	3,5	
	10	< 100, 3 wks, 48, ear tag				
	53 (1)	0 (0)	4,6	no evaluation of batch 1–10 due to technical problems with the device		
	14	< 100, 2 wks, 24, iron, vaccination	19 (1)	125 (5)	2,3	No evaluation of batch 6,7,8,9,10 due to technical problems with the device
3 devices	15 farms		**1568 (15)**	**11,574 (129)**		**30 batches**

The numbers in bold also show the total number of used devices, farms, piglets and batches involved

farm size: Ø n sow, farrowing rhythm in weeks, piglet/batch, routine management procedures on castration day

*AF* Anaesthetic free castration in 2020; *IA* Castration under isoflurane anaesthesia; *iron* Iron supplementary, *Ear tag* Ear tagging, *tail dock* Tail docking; *wks* weeks

## Study design

For this purpose, on each farm, the castration procedure was surveyed in one farrowing batch without anaesthesia (AF) in 2020 and ten consecutive farrowing batches where castration was performed under isoflurane anesthesia (IA) in 2021. In each farrowing batch, piglet losses and duration of castration process itself as well as the number of sows and castrated piglets were obtained. Moreover, defensive movements and anaesthetic incidents of the piglets during castration under isoflurane anaesthesia and isoflurane consumption were recorded. The complete working time was measured in the AF batch and three IA batches and also, isoflurane exposure was assessed in two IA batches per farm. In total, data from 11,574 piglets from 129 IA farrowing batches with an average age of 4.94 ± 0.9 days were collected and compared to data from 1568 piglets of 15 AF batches with a mean age of 5.0 ± 1.2 days in 2020. In four batches (one AF and three IA batches) per farm, data collection took place in presence of the Clinic of Swine (CFS). On castration days, the collection of data began with the preparation of the necessary utensils. In IA batches, the anaesthetic devices had to be set up and put into operation. Therefore, all male piglets were administered analgesic treatment (Meloxicam 0,4 mg/kg, Metacam^®^ 5 mg/ml i. m., Boehringer Ingelheim Vetmedica GmbH, Ingelheim/Rhein) at least 30 min before castration and additionally internal management procedures (e.g. ear tagging, iron supplementation, vaccination programme, tail docking) were carried out (Table 3). The farmers used their individual work flows to carry out castration process without and under isoflurane anaesthesia. Subsequently, castration procedure started in AF batches without anaesthesia and in IA batches under isoflurane anaesthesia. For IA, three types of anaesthetic devices (PigNap 4.0 BEG Schulze Bremer GmbH, PorcAnest 3000 Promatec Automation AG, Anestacia GDO Precision Technology GmbH) were used. All three are working with 5 Vol % isoflurane (Isofluran Baxter vet, 1000 mg/g, Baxter Deutschland GmbH, Unterschleißheim) mixed with room air as carrier gas and the narcotic gas was released for 70–90 s over the provided masks. After fixation in the anaesthetic device and expiry of the initiation time, the farmers had to check the interdigital claw reflex to assure sufficient anaesthetic depth. If there was a reaction, the anaesthesia had to be prolonged. Consistently with AF piglet castration, castration under IA was performed by small skin incision, forward shifting of the testicles and serving the spermatic cord by using a scalpel (AF) or emasculator (IA). After castration, piglets were removed from the fixation (AF) or the inhalation masks (IA) and replaced in a Box to wake up (IA). As soon as they could stand on their own, the piglets were replaced to the sows. Due to malfunction of the anaesthetic devices on two farms [7, 14] only four respectively five batches could be evaluated. Although all data from farm 10 were collected, they couldn´t be analysed due to persistent technical problems (Table 3). Moreover, IA batches on farm 3 (PA), 2, 6, 11 (PN) had to be stopped and repeated due to technical problems which occurred on the castration day.

## Anaesthetic devices and castration process under isoflurane anaesthesia (IA)

The anaesthetic devices of the three brands differed in various characteristics. The PigNap 4.0 is equipped with four operating units and the evaporator has a heater. The initiation time is 70 s. After placing the piglets backwards into the provided double-walled masks, the narcotic gas-air-mixture is inhaled from a breathing bag. For each piglet there is a separate control light which triggers a magnetic plunger by pressing the trunk disc on a valve, whereby the gas mixture is actively inhaled through the breathing bag. This ensures the individual needs (e.g. size of piglets) of each piglet. After 55 s the control light starts flashing, after another 15 s the colour changes from red to green, which signals the start of castration. If the depth of anaesthesia is insufficient, gas flow must be prolonged and should be completed after 120 s after the piglet is placed [28]. The PorcAnest 3000 has three operating units and a heater as well. The piglets are fixed in the holder on their bellies and by pressing the trunk on the sieve gland, the electromagnetic valve opens. Before the initiation time of 90 s expires, the piglets are turned onto their back. After 70 s the counter starts to blink, which signals the start of the castration. As long as the pressure on the mask remains, the gas supply is maintained but after 90 s the active gas flow for the respective station is switched off. If necessary, a new anaesthetic cycle must be started if the depth of anaesthesia is insufficient, by replacing the piglet in the mask [29]. The Anestacia device was used with three and four operating units, all devices were equipped with a heater. After securing the piglets the photoelectric sensor detects the inserted piglet and the timer starts counting down from 85 to 15 s. During that time, the blue LED “Isoflurane On” lights up. After 70 s, the green LED “Castration” turns on. As soon as the pre-set time of 15 s has counted down to zero, the isoflurane automatically switches of and the fresh air supply starts. It is possible to extend the duration of anaesthesia once for all units for 30 s [30].

## Data collection

### Defensive movements

For data sampling, the castration process of all male piglets was recorded by video camera including four farrowing batches under supervision of the CFS and seven farrowing batches by the farmer. Afterwards, the recorded videos were analysed always by the same person. Defensive movements were evaluated during castration process in IA batches due to the intensity of movement and vocalisation and rated by using a score system as shown in Table 4 [8, 11]. In the evaluation, score 0 and 1 were combined and represent sufficient depth of anaesthesia. Also, score 3 and 4 were combined and represent insufficient depth of anaesthesia [8, 12]. In addition, it was investigated whether the location of the device (stable aisle and farrowing compartment) has an impact on the depth of anaesthesia.

**Table 4:** Modified movement score after Wenger et al. [11], and Härtel et al. [8]

Score	Defensive movements/Vocalisation
0	No defensive movements
1	1 short defensive movement of one limp, no vocalisation
2	2 short defensive movements, no vocalisation
3	3–4 short defensive movements or 1 long defensive movement or one vocalisation
4	> 4 short or > 1 long defensive movement or > one vocalisation

### Anaesthetic incidents and mortality rate

If anaesthetic incidents (apnea, cardiovascular arrest, gasp) occurred during the anaesthetic or recovery phase, appropriate countermeasures (panning, cold water pouring, and cardiac massage) were initiated and documented in a pre-made sheet. Additionally, the piglet mortality rate after the castration process until 24 h were assessed.

### Labour time and isoflurane consumption

To determine isoflurane consumption and labour time under isoflurane anaesthesia, data were obtained over three batches on each farm. The required working time for the complete castration process “complete process” and also for the individual process steps “preparation” (required material, set up of the device in IA batches), “analgesia” (administration of the analgesic) and “post-processing” (cleaning, disinfection and disassembly of the device in IA batches) was collected in three IA and the one AF batch per farm with a chronometer in minutes (min) by the CFS. The individual process step “castration” (start of first piglet until last piglet) was measured afterwards according to the video records for each batch. The time for “complete process”, “analgesia” and “castration” was corrected by the number of castrated piglets per batch. Due to technical problems with the device and the termination of the castration process under IA on farm 10, only 45 IA batches were evaluated. For further evaluation of economic effects, the farmer was asked to perform the complete castration in IA batches and also in AF batch with the same number of persons. The number of people involved varied between the farms (two and three persons), but did not vary within the farms over the entire test period. As the collected required working time was determined regardless of the number of persons involved, the workload was calculated considering the required labour time for “complete process” x number of persons involved deducting idle times of individual persons (Additional file 1: Table S1). Isoflurane consumption was calculated due to filled and used isoflurane and corrected by the number of castrated piglets per batch. It was also documented by the CFS on three farm visits.

### Isoflurane exposure

The sampling and analysis of isoflurane exposure concentration in the ambient air was carried out by TÜV SÜD Industrie Service GmbH according to the procedure of Härtel et al. [8] and IFA protocol no. 7673 (10/2004) twice on 15 farms. Unfortunately, two farms (7, 10) had to be excluded from the evaluation, due to persistent technical problems with their devices. The measurements included five stations: in the ambient air of the castrating person, the ambient air of the person transporting the piglet crates, the ambient air of the anaesthesia mask, on the activated charcoal filter and in the piglet boxes. In accordance with the Ordinance on Hazardous Substances, the usual occupational exposure limit value (OEL) was determined. A shift mean value describing the exposure on 5 days per week for 8 h each, was extrapolated to the lifetime working hours [31].

## Statistical analysis

The collected data was summarised in a database using Microsoft Excel^®^ 2019 (Fa. Microsoft, Redmond, USA) and analysed statistically in IBM SPSS^®^ Statistics Version 26.0 (Fa. IBM Corp. Armonk, USA). Labour time, workload, isoflurane consumption and isoflurane exposition data were descriptively and presented as mean and standard deviation. In order to test the non-normally distributed parameters for significance (isoflurane consumption, workload and required working time separated after devices and IA/AF batches) a Kruskal–Wallis-Test was performed. For comparing required time between IA and AF batches a Mann–Whitney-U test was performed.

For analysing proportion of animals with sufficient depth of anaesthesia and anaesthetic incidences per batch, all data wrangling and statistical analyses were conducted using R Statistical language (version 4.0.3; R Core Team, 2020). A total of 28 missing values (14 in depth of anaesthesia and 14 in incidents of anaesthesia) were imputed via the missRanger approach, a non-parametric multivariate imputation by chained random forest algorithm with 1000 trees [32]. This method combines random forest imputation [33, 34] with predictive mean matching [35] and thus iterates multiple times until the average out-of-bag prediction error of the models stops to improve. The quality of imputation was then controlled visually and since all imputed values fell into the distribution of the existing values, the result of the imputation was accepted. Generalized Additive Models for Location Scale and Shape were used to model both the percentages of depth of anaesthesia and percentages of incidents of anaesthesia on farms. One-inflated beta distribution family was used to model the percentages of depth of anaesthesia, since it included numerous ones (100%). Zero-inflated beta distribution family was used to model the percentages of incidents of anaesthesia, since it included multiple zeros (0%). All contrasts (differences) between particular categories of categorical variables were assessed after model-fitting by the estimated least-squares marginal means (emmeans) with the Tukey *p*-value correction for multiple comparisons. Results with a *p*-value < 0.05 were considered statistically significant, while results with a *p*-value < 0.1 were considered suggestive.

## Supplementary Information

**Additional file1: Table S1** Workload (in minutes) for complete process per piglets divided into IA and AF batches per device.

**Additional file2: Table S2** Results of the isoflurane concentration measurements (TÜV SÜD).

## Data Availability

The datasets used and analysed during the current study are not publicly available due to certain restrictions concerning confidentiality but are available from the corresponding author on reasonable request.

## Abbreviations

AF: Anaesthetic free
AN: Anestacia device
CFS: Clinic for swine
DLG: German agricultural society
IA: Isoflurane anaesthesia
min: Minimum
max: Maximum
n: Number
PA: PorcAnest device
PN: PigNap device
SD: Standard derivation
TÜV: TÜV, SÜD AG, Technical monitoring association
