# Supplementary material for: Implementation of piglet castration under inhalation anaesthesia on farrowing farms
Source: Porcine Health Manag. 2022 May 17;8:20. doi: 10.1186/s40813-022-00263-0 (PMC9115960; doi:10.1186/s40813-022-00263-0)
Supplement: Supplementary file 1 — Additional file1: Table S1 Workload (in minutes) for complete process per piglets divided into IA and AF batches per device. [file 40813_2022_263_MOESM1_ESM.pdf]

device	workload (minutes)					
	IA batches			AF batches		
	n	mean ± SD	min/max	n	mean ± SD	min/max
Complete process*	45	4.5 ± 1.7 [b]	1.5 - 9.1	14	3.0 ± 1.6[a]	1.5 - 7.2
PN	15	3.5 ± 1.5[A]	2.1 - 6.9			
PA	18	4.4 ± 0.9[A,B]	2.8 - 6.0			
AN	12	5.6 ± 2.1[B]	1.5 - 9.1			

*: complete castration process per piglet
a,b: differs significantly within a row
A,B: differs significantly within a column
