# Supplementary material for: Implementation of piglet castration under inhalation anaesthesia on farrowing farms
Source: Porcine Health Manag. 2022 May 17;8:20. doi: 10.1186/s40813-022-00263-0 (PMC9115960; doi:10.1186/s40813-022-00263-0)
Supplement: Supplementary file 2 — Additional file2: Table S2 Results of the isoflurane concentration measurements (TÜV SÜD). [file 40813_2022_263_MOESM2_ESM.pdf]

Farm ID	device	piglet boxes	castration		transport		mask		carbon filter		pigletbox	
			1[a]	2[b]	1	2	1	2	1	2	1	2
2	**PN**	1[c]	5.6[e]	4.4	8.2	4.7	13.3	40.2	-	4.4	64.0	32.7
6		1	1.8	1.5	-	-	48.6	45.5	4.8	3.1	24.4	29.8
11		1	4.2	2.8	3.5	2.2	13.3	15.7	7.8	4.8	87.6	41.1
12		2[d]	7.4	1.6	6.6	1.6	12.3	129.0	9.6	3.6	126.3	142.9
13		2	7.8	0.6	9.6	5.2	41.6	5.5	7.5	0.4	31.8	27.41
1	**PA**	2	1.7	0.8	4.7	3.4	16.2	15.9	60.7	8.4	16.3	156.1
3		2	3.8	3.3	4.4	2.8	8.4	10.0	7.3	2.8	23.1	96.6
4		1	20.3	6.6	10.7	3.6	51.1	48.3	7.7	1.4	84.3	58.1
5		2	1.0	1.2	2.9	4.5	14.5	20.7	0.2	0.6	69.6	177.8
15		2	23.3	7.4	6.6	3.4	88.4	30.4	8.2	3.4	210.9	268.9
7	**AN**	2	-	-	-	-	-	-	-	-	-	-
8		2	8.7	8.1	5.2	4.7	48.2	16.6	8.1	7.4	80.6	57.4
9		2	2.2	9.3	2.1	2.7	66.0	75.1	0.8	9.4	207.3	34.7
10		2	-	-	-	-	-	-	-	-	-	-
14		2	6.3	8.7	5.7	4.0	7.6	17.5	4.6	6.9	50.3	93.5

[a] 1st measurement

[b] 2nd measurement

[c] opened piglet boxes

[d] closed piglet boxes

[e] mg/m^3
